# Two-component systems required for virulence in *Pseudomonas aeruginosa*

**DOI:** 10.1093/femsle/fnx104

**Published:** 2017-05-16

**Authors:** Vanessa I. Francis, Emma C. Stevenson, Steven L. Porter

**Affiliations:** Biosciences, Geoffrey Pope Building, College of Life and Environmental Sciences, University of Exeter, Exeter EX 4QD, UK

**Keywords:** Two-component signalling, multikinase network, *Pseudomonas aeruginosa*, secondary messengers, virulence

## Abstract

*Pseudomonas aeruginosa* is a versatile opportunistic pathogen capable of infecting a broad range of hosts, in addition to thriving in a broad range of environmental conditions outside of hosts. With this versatility comes the need to tightly regulate its genome to optimise its gene expression and behaviour to the prevailing conditions. Two-component systems (TCSs) comprising sensor kinases and response regulators play a major role in this regulation. This minireview discusses the growing number of TCSs that have been implicated in the virulence of *P. aeruginosa*, with a special focus on the emerging theme of multikinase networks, which are networks comprising multiple sensor kinases working together, sensing and integrating multiple signals to decide upon the best response. The networks covered in depth regulate processes such as the switch between acute and chronic virulence (GacS network), the Cup fimbriae (Roc network and Rcs/Pvr network), the aminoarabinose modification of lipopolysaccharide (a network involving the PhoQP and PmrBA TCSs), twitching motility and virulence (a network formed from the Chp chemosensory pathway and the FimS/AlgR TCS), and biofilm formation (Wsp chemosensory pathway). In addition, we highlight the important interfaces between these systems and secondary messenger signals such as cAMP and c-di-GMP.

## Teaser

Two-component systems controlling the virulence of the opportunistic pathogen, *Pseudomonas aeruginosa*.

## INTRODUCTION

*Pseudomonas aeruginosa* has a remarkably diverse ability to thrive in many different environments both outside and within a host. To be successful in these diverse situations, *P. aeruginosa* needs to sense its environment, decide upon an appropriate response and modify its behaviour accordingly to better suit prevailing conditions. Regulatory networks are key to this decision-making process. *Pseudomonas aeruginosa* has a large genome (6.3 Mb for the reference PAO1 strain), reflecting the diverse range of environments and hosts that it can inhabit, and just under 10% of its genes are dedicated to these regulatory networks (Stover *et al.*^2000^). Two-component systems (TCSs) comprising sensor kinases (SKs) and response regulators (RRs) (Stock, Robinson and Goudreau 2000) play a major role in these regulatory networks with *P. aeruginosa* having 64 SKs, 72 RRs and 3 Hpt proteins (Rodrigue *et al.*^2000^; Stover *et al.*^2000^).

As an opportunist pathogen, being capable of both acute and chronic infection, *P. aeruginosa* has a multitude of virulence factors and antibiotic resistance determinants (Driscoll, Brody and Kollef 2007; Gooderham and Hancock 2009; Coggan and Wolfgang 2012). Well over 50% of the TCSs of *P. aeruginosa* have been linked to virulence, controlling either virulence-related behaviour or contributing towards *in vivo* fitness and colonisation ability. This number has grown considerably in recent years, primarily due to the successful application of whole-genome-based methodologies for identifying genes involved in virulence, such as Tn-Seq approaches using animal infection models, and the study of pathoadaptive mutations in isolates from cystic fibrosis (CF) patients (Table 1 major).

**Table 1.** The TCSs that have been implicated in *P. aeruginosa* virulence and/or antibiotic resistance.

Sensor kinase		Response regulator												
PAO1	PA14	PAO1	PA14	Protein product	Signalling molecule	Functional description	Chronic (Potvin *et al.*^2003^)	Pathoadaptive (Marvig *et al.*^2013^)	Pathoadaptive (Marvig *et al.*^2015^)	Fitness Tn-Seq (Skurnik *et al.*^2013^)	Acute burn model (Turner *et al.*^2014^)	Chronic wound model (Turner *et al.*^2014^)	CF sputum Tn-Seq (Turner *et al.*^2015^)	References
Multikinase networks														
^a^GacS network controlling the acute/chronic switch														
PA0928	PA14_52260	PA2586	PA14_30650	GacS-GacA	Solvent extractable extracellular signal	GacA–GacS system. Virulence, quorum-sensing-dependent regulation of exoproducts and virulence factors, biofilm formation, antibiotic resistance, swarming motility, iron metabolism and T3/T6 secretion					Y	Y		Reimmann *et al.* (1997); Rahme *et al.* (2000); Parkins, Ceri and Storey (2001); Heeb, Blumer and Haas (2002); Goodman *et al.* (2004); Soscia *et al.* (2007); Brencic *et al.* (2009); Goodman *et al.* (2009); Frangipani *et al.* (2014)
PA1611	PA14_43670				Unknown	PA1611-HptB-HsbR phosphorelay. Acute/chronic infection cycle in conjunction with the GacS network and has been shown to directly interact with RetS						Y	Y	Lin *et al.* (2006; Hsu *et al.* (2008); Kong *et al.* (2013); Bhagirath *et al.* (2017)
PA2824	PA14_27550			SagS	Unknown	Regulates the motile-sessile switch in biofilm formation. Linked with the GacS and HptB networks and the SK BfiS						Y		Hsu *et al.* (2008); Petrova and Sauer (2010, 2011)
PA4197	PA14_09680	PA4196	PA14_09690	BfiS-BfiR	Unknown	Biofilm formation/maintenance				Y	Y	Y		Petrova and Sauer (2009)
PA3345	PA14_20800	PA3346	PA14_20780	HptB-HsbR	Unknown	HptB-mediated phosphorelay, swarming motility and biofilm formation		Y				Y	Y	Hsu *et al.* (2008); Bhuwan *et al.* (2012)
PA3974				LadS	Ca^2+^	Regulates virulence, biofilm formation and T3 secretion/cytotoxicity via GacS					Y	Y		Ventre *et al.* (2006); Chambonnier *et al.* (2016)
PA4856	PA14_64230			RetS	Kin cell lysate	Regulates virulence, biofilm formation and T3/T6 secretion/cytotoxicity via GacS			Y		Y	Y		Goodman *et al.* (2004); Laskowski, Osborn and Kazmierczak (2004); Moscoso *et al.* (2011); LeRoux *et al.* (2015)
^a^Roc network controlling the fimbrial *cup* genes														
PA3044	PA14_24720	PA3045	PA14_24710	RocS2-RocA2	Unknown	RocA2–RocS2 system. Regulation of fimbriae adhesins and antibiotic resistance					Y	Y	Y	Kulasekara *et al.* (2005); Sivaneson *et al.* (2011)
PA3946	PA14_12820	PA3947 PA3948	PA14_12810PA14_12780	RocS1 (SadS)-RocR (SadR) RocA1 (SadA)	Unknown	RocS1–RocR–RocA1 (SadA–SadR–SadS system). Biofilm maturation, fimbrial genes, T3 secretion and antibiotic resistance. RocA1 contains EAL output domain, RocR is a RocA1 antagonist					Y			Gallagher and Manoil (2001); Kuchma, Connolly and O’Toole (2005); Kulasekara *et al.* (2005); Sivaneson *et al.* (2011)
^a^RcsCB/PvrSR network controlling the *cupD* fimbrial genes														
	PA14_59800		PA14_59790	PvrS-PvrR	Unknown	Phenotypic variation, antibiotic resistance, biofilm formation. Controls *cupD* fimbriae genes								Drenkard and Ausubel (2002); Mikkelsen *et al.* (2009, 2013)
	PA14_59780		PA14_59770	RcsC-RcsB	Unknown	Biofilm formation. Controls *cupD* fimbriae genes								Mikkelsen *et al.* (2009, 2013)
Network controlling ethanol oxidation														
PA1976/PA1979	PA14_38970 PA14_38910	PA1978 PA1980	PA14_38930 PA14_38900	ErcS'/EraS-ErbR/EraR	Possible cytosolic metabolites	Regulates ethanol oxidation control and it is implicated in biofilm specific antibiotic resistance. PA14_38910 is essential		Y		Y	Y	Y	Y	Mern *et al.* (2010); Beaudoin *et al.* (2012)
PA1992	PA14_38740			ErcS	Possible cytosolic metabolites	Regulates ethanol oxidation control and it is implicated in biofilm specific antibiotic resistance							Y	Mern *et al.* (2010); Beaudoin *et al.* (2012)
		PA3604	PA14_17670	ErdR	Unknown	Ethanol oxidation control, implicated in biofilm-specific antibiotic resistance					Y			Mern *et al.* (2010); Beaudoin *et al.* (2012)
Network detecting phosphate limitation and tricarboxylic acids														
PA0757	PA14_54500	PA0756	PA14_54510	TctE-TctD	Tricarboxylic acids	Controls expression of tricarboxylic acid uptake system					Y	Y	Y	Bielecki *et al.* (2015)
PA5361	PA14_70760	PA5360	PA14_70750	PhoR–PhoB	Inorganic phosphate	Quorum sensing and swarming motility					Y	Y		Blus-Kadosh *et al.* (2013); Faure *et al.* (2013); Bielecki *et al.* (2015)
^a^Chp/FimS/AlgR network controlling twitching motility, virulence and biofilm formation														
PA0413	PA14_05390	PA0408 PA0409 PA0414	PA14_05320 PA14_05330PA14_05400	ChpA/PilG/ PilH/ChpB	Unknown	Chemosensory pili (Pil–Chp) system, twitching motility and cAMP levels. Virulence genes					Y	Y		Darzins and Russell (1997); Whitchurch *et al.* (2004); Bertrand *et al.* (2010); Fulcher *et al.* (2010); Luo *et al.* (2015); Persat *et al.* (2015); Inclan *et al.* (2016); Silversmith *et al.* (2016)
PA5262	PA14_69480	PA5261	PA14_69470	FimS(AlgZ)-AlgR	Unknown	Virulence, alginate biosynthesis, twitching and swarming motility, biofilm formation, cyanide production, cytotoxicity and type III secretion system gene expression				Y	Y	Y		Intile *et al.* (2014); Okkotsu, Little and Schurr (2014)
^a^Network controlling the aminoarabinose modification of LPS														
PA1179	PA14_49170	PA1179	PA14_49180	PhoQ–PhoP	Mg^2+^	Low Mg^2+^ signal. Polymyxin, antimicrobial peptide and aminoglycoside resistance. Virulence, swarming motility and biofilm formation					Y	Y		Ernst *et al.* (1999); Macfarlane *et al.* (1999); Macfarlane, Kwasnicka and Hancock (2000); Ramsey and Whiteley (2004); McPhee *et al.* (2006); Jochumsen *et al.* (2016)
PA1798	PA14_41270	PA1799	PA14_41260	ParS-ParR	Cationic peptides	Multidrug resistance, quorum sensing, phenazine production and swarming						Y		Fernández *et al.* (2010); Muller, Plésiat and Jeannot (2011); Wang *et al.* (2013)
PA3078	PA14_24340	PA3077	PA14_24350	CprS-CprR	Antimicrobial peptides	Triggers LPS modification and adaptive antimicrobial peptide resistance				Y				Fernández *et al.* (2010)
PA4380	PA14_56940	PA4381	PA14_56950	ColS-ColR	Zn^2+^	Polymyxin resistance, mutants have decreased virulence in a *C*aenorhabditis *elegans* model and decreased cell adherence					Y	Y	Y	Garvis *et al.* (2009); Gutu *et al.* (2013)
PA4777	PA14_63160	PA4776	PA14_63150	PmrB–PmrA	Mg^2+^	Induced by low Mg^2+^ and cationic antimicrobial peptides. Polymyxin B, colistin and antimicrobial peptide resistance		Y				Y	Y	McPhee, Lewenza and Hancock (2003); Moskowitz, Ernst and Miller (2004); McPhee *et al.* (2006); Lee and Ko (2014)
Other TCSs implicated in virulence														
PA0033	PA14_00420			HptC	Unknown	Histidine containing phosphotransfer protein					Y	Y		
		PA0034	PA14_00430		Unknown	PA0034 is repressed during *in vitro* growth in CF sputum medium. Located directly upstream of *hptC* (PA0033)					Y	Y		Palmer *et al.* (2005)
		PA0173	PA14_02180	CheB	Unknown		Y					Y	Y	
PA0178	PA14_02250	PA0179	PA14_02260		Unknown						Y	Y		
PA0991	PA14_51480			HptA	Unknown	Histidine containing phosphotransfer protein					Y	Y		
PA0464	PA14_06070	PA0463	PA14_06060	CreC–CreB	Penicillin-binding protein 4	Catabolism. Swarming and swimming motility. Antibiotic resistance, biofilm and global gene regulation					Y	Y		Wagner *et al.* (2007); Zamorano *et al.* (2014)
PA0600	PA14_07820	PA0601	PA14_07840	AgtS-AgtR	Peptidoglycan	Involved in sensing peptidoglycan and controlling virulence					Y	Y	Y	Korgaonkar *et al.* (2013)
PA0930	PA14_52240	PA0929	PA14_52250	PirR–PirS	Unknown	Iron acquisition	Y				Y	Y	Y	Vasil and Ochsner (1999)
PA1098	PA14_50200	PA1099	PA14_50180	FleS–FleR	Unknown	Flagellar motility and adhesion to mucin. FleS likely cytoplasmic sensor					Y	Y		Ritchings *et al.* (1995); Dasgupta *et al.* (2003)
PA1136	PA14_46980	PA1135	PA14_49710		Unknown	Antibodies against PA1136 found in CF patient sera								Beckmann *et al.* (2005)
PA1158	PA14_49420	PA1157	PA14_49440		Unknown		Y				Y	Y	Y	
PA1243	PA14_48160				Unknown							Y	Y	
PA1336	PA14_46980	PA1335	PA14_46990	AauS-AauR	Unknown						Y			
PA1396	PA14_46370	PA1397	PA14_46360		DSF	Interspecies signalling. Responds to diffusible signal factor (DSF) and regulates biofilm formation and antibiotic resistance				Y				Ryan *et al.* (2008)
PA1438	PA14_45870	PA1437	PA14_45880		Unknown						Y			
		PA1456	PA14_45620	CheY	Unknown					Y	Y	Y		
PA1458	PA14_45590	PA1459	PA14_45580		Unknown						Y	Y		
PA1636	PA14_43350	PA1637	PA14_43340	KdpD-KdpE	Unknown						Y	Y		
		PA1785	PA14_41490	NasT	Unknown					Y	Y	Y		
		PA2137	PA14_36920		Unknown						Y	Y		
PA2177	PA14_36420				Unknown						Y	Y	Y	
		PA2376	PA14_33920		Unknown						Y	Y	Y	
PA2480	PA14_32570	PA2479	PA14_32580		Unknown	Essential in PA14				Y	Y			
PA2524	PA14_31950	PA2523	PA14_31960	CzcS–CzcR	Zinc, cadmium or cobalt	Regulates metal resistance and antibiotic resistance and pathogenicity					Y			Hassan *et al.* (1999); Dieppois *et al.* (2012)
PA2571	PA14_30840	PA2572	PA14_30830		Unknown	Affects motility, virulence and antibiotic resistance. Works with PA2573 (an MCP homologue)						Y		McLaughlin *et al.* (2012)
PA2583	PA14_30700				Unknown						Y			
PA2656	PA14_29740	PA2657	PA14_29730	BqsS-BqrR/(CarS-CarR)	Extracellular Fe(II) and CaCl_2_	Biofilm decay, ferrous iron sensing, antibiotic resistance and cationic stress tolerance. Maintains Ca^2+^ homeostasis, regulates pyocyanin, swarming and tobramycin sensitivity. PA14_29740 is an essential gene				Y	Y	Y	Y	Dong *et al.* (2008); Kreamer, Costa and Newman (2015); Guragain *et al.* (2016)
PA2687	PA14_29360	PA2686		PfeS–PfeR	Enterobactin	Iron acquisition					Y	Y		Dean, Neshat and Poole (1996)
		PA2798	PA14_27940		Unknown	Described as essential in PA14				Y	Y	Y		
PA2810	PA14_27800	PA2809	PA14_27810	CopS–CopR	Copper	Metal and imipenem resistance				Y	Y	Y		Teitzel *et al.* (2006); Caille, Rossier and Perron (2007)
PA2882	PA14_26810	PA2881	PA14_26830		Unknown						Y	Y		
		PA2899	PA14_26570		Unknown							Y		
PA3191	PA14_22960	PA3192	PA14_22940	GtrS-GltR	2-Ketogluconate	Glucose transport and type III secretion cytotoxicity	Y			Y	Y	Y	Y	Wolfgang *et al.* (2003); O’Callaghan *et al.* (2012); Daddaoua *et al.* (2014)
PA3206	PA14_22730	PA3204	PA14_22760	CpxA-CpxR	Unknown	Antibodies against PA3206 found in CF patient sera. Implicated in cell envelope stress response. Activates MexAB-OprM efflux pump expression and enhances antibiotic resistance				Y	Y	Y		Beckmann *et al.* (2005); Yakhnina, McManus and Bernhardt (2015); Tian *et al.* (2016)
PA3271	PA14_21700				Unknown			Y				Y		
		PA3349	PA14_20750		Unknown						Y			
PA3462	PA14_19340				Unknown							Y		
PA3704	PA14_16470	PA3702	PA14_16500	WspE–WspR	Surface-associated growth	Wsp chemosensory system. Regulates biofilm, autoaggregation and cyclic-di-GMP. WspR contains GGDEF output domain, WspE is CheA-type sensor			Y		Y	Y		D’Argenio *et al.* (2002); Hickman, Tifrea and Harwood (2005); Kulasekara *et al.* (2005); Borlee *et al.* (2010); Huangyutitham *et al.* (2013)
		PA3714	PA14_16350		Unknown						Y			
PA3878	PA14_13740	PA3879	PA14_13730	NarX–NarL	Nitrate	Nitrate sensing and respiration. Biofilm formation, fermentation, swimming and swarming motility		Y			Y			Van Alst *et al.* (2007); Benkert *et al.* (2008)
		PA4032	PA14_11680									Y		
PA4036	PA14_11630				Unknown							Y		
		PA4080	PA14_11120		Unknown						Y	Y		
PA4102		PA4101		BfmS-BfmR	Unknown	Biofilm formation/maintenance					Y	Y	Y	Petrova and Sauer (2009)
PA4112	PA14_10770				Unknown	Antibodies against this protein found in CF patient sera		Y			Y	Y		Beckmann *et al.* (2005)
PA4293	PA14_55780	PA4296	PA14_55810	PprA–PprB	Unknown	Outer membrane permeability and aminoglycoside resistance. Virulence including T3 secretion and biofilm formation		Y				Y	Y	Wang *et al.* (2003); Giraud *et al.* (2011); de Bentzmann *et al.* (2012)
PA4398	PA14_57170	PA4396	PA14_57140		Unknown	Overexpression impairs T3 secretion-mediated cytotoxicity. GGDEF output domain. In PA14, PA4398 sensor kinase regulates motility and biofilm. PA14_57170 is essential in PA14				Y	Y			Kulasakara *et al.* (2006); Strehmel *et al.* (2015)
PA4494	PA14_58320	PA4493	PA14_58300	RoxS-RoxR	Possibly cyanide	Cyanide tolerance. Neutrophil transmigration response				Y	Y	Y		Comolli and Donohue (2002); Hurley *et al.* (2010); Fernández-Piñar *et al.* (2012)
PA4546	PA14_60250	PA4547	PA14_60260	PilS–PilR	Pilin subunits	Biofilm formation, type IV pilus expression, twitching and swarming motility				Y	Y	Y	Y	Ishimoto and Lory (1992); Hobbs *et al.* (1993); Overhage *et al.* (2007); Kilmury and Burrows (2016)
PA4725	PA14_62530	PA4726	PA14_62540	CbrA–CbrB	Various carbon sources	Carbon and nitrogen storage, cytotoxicity, swarming motility, modulates metabolism, virulence and antibiotic resistance in PA14					Y	Y	Y	Gallagher and Manoil (2001); Rietsch, Wolfgang and Mekalanos (2004); Wagner *et al.* (2007); Yeung, Bains and Hancock (2011); Yeung *et al.* (2014)
		PA4781	PA14_63210		Unknown						Y			
PA4886	PA14_64580	PA4885	PA14_64570	IrlR	Unknown		Y			Y	Y	Y		
		PA4959	PA14_65540	FimX	Unknown	Phosphodiesterase (GGDEF and EAL domains). Signal transduction protein involved in twitching motility phosphotransfer activity, and cyclic di-GMP metabolism. Reduced *in vitro* cytotoxicity								Huang, Whitchurch and Mattick (2003); Kazmierczak, Lebron and Murray (2006); Kulasakara *et al.* (2006); Jain *et al.* (2012)
PA4982	PA14_65860	PA4983	PA14_65880	AruS-AruR	Arginine	Antibodies against this protein found in CF patient sera. Controls expression of the arginine transaminase pathway					Y			Beckmann *et al.* (2005); Yang and Lu (2007)
PA5124	PA14_67670	PA5125	PA14_67680	NtrB-NtrC	PII—nitrogen status	Responds to cellular nitrogen levels and activates nitrogen scavenging genes					Y	Y	Y	Li and Lu (2007)
PA5165	PA14_68230	PA5166	PA14_68250	DctB-DctD	C4-dicarboxylates	Controls expression of C4-dicarboxylate transporters				Y	Y	Y	Y	Valentini, Storelli and Lapouge (2011)
PA5199	PA14_68680	PA5200	PA14_68700	AmgS-AmgR	Aminoglycosides	Aminoglycoside resistance and cell envelope stress response. Described as essential in PA14				Y	Y	Y		Lau *et al.* (2013, 2015)
														
		PA5364	PA14_70790		Unknown							Y		
PA5484	PA14_72390	PA5483	PA14_72380	KinA-AlgB	Unknown	Alginate biosynthesis. Virulence, acute/chronic switch					Y	Y	Y	Leech *et al.* (2008); Chand *et al.* (2011); Chand, Clatworthy and Hung (2012)
PA5512	PA14_72740	PA5511	PA14_72720	MifS-MifR	α-Ketoglutarate	Biofilm formation and metabolism						Y		Tatke *et al.* (2015)

The TCSs known to form multikinase networks are listed in the first section and the others are listed in the second section. The columns to the right of the description column refer to whole genome studies investigating virulence using the following methodologies: Tn-Seq, signature tagged mutagenesis, and the study of pathoadaptive mutations in CF patient isolates. ‘Y’ indicates that the study has implicated the TCS in virulence.

a Highlights the five multikinase networks that are discussed in depth in this minireview.

TCSs are generally considered to work alone, sensing either a single stimulus or a narrow range of stimuli to control appropriate responses, being insulated from significant crosstalk (Laub and Goulian 2007; Capra *et al.*^2012^), with relatively few exceptions (Willett and Crosson 2017). However, a recently emerging theme, in which tremendous progress has been made in the last few years, is the discovery that multikinase networks play leading roles in orchestrating the virulence of *P. aeruginosa*. Multikinase networks comprise multiple SKs that collaborate to form sophisticated networks capable of sensing and integrating multiple stimuli. In the following sections, we explore how these networks regulate virulence.

## THE TRANSITION BETWEEN ACUTE AND CHRONIC MODES OF INFECTION: THE GacS NETWORK

**Figure 1.**
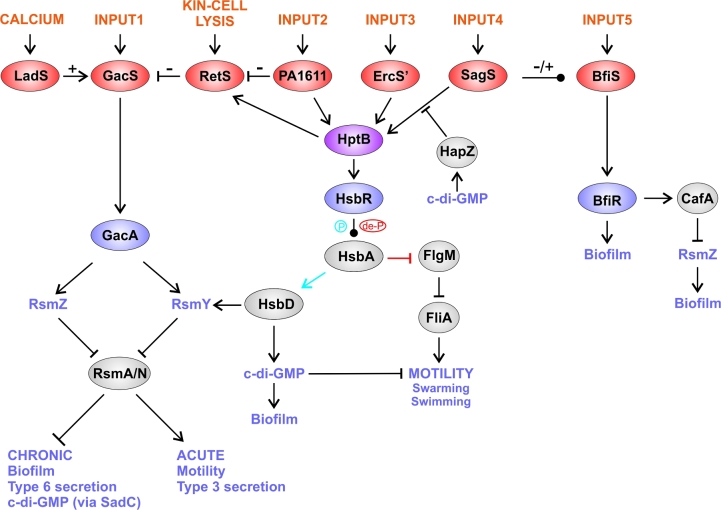
The GacS network including the closely affiliated HptB and SagS/BfiS branches. Red ovals show SKs, blue ovals show RRs, the purple oval shows the HptB protein and the grey ovals show other proteins in the system. Arrows show stimulatory interactions, while blunt-ended lines show inhibitory interactions and bulb-ended lines show interactions that can be stimulatory or inhibitory depending on conditions. The primary output of the GacS side of the pathway is the small RNAs RsmY and RsmZ, which sequester the post-transcriptional regulators, RsmA and RsmN. When RsmA and RsmN are sequestered, virulence genes associated with chronic infection are upregulated while those associated with acute virulence genes are downregulated. Conversely, when RsmA and RsmN are free, the acute virulence genes are upregulated and the chronic infection genes are downregulated. The HptB and SagS/BfiS branches of the pathway also regulate RsmY and RsmZ levels, respectively. The role of HsbA differs depending on whether it is phosphorylated (blue arrow) or dephosphorylated (red arrow). Two diguanylate cyclases are controlled by this network, HsbD and SadC.

The GacS network plays a leading role in governing the transition between acute and chronic modes of infection. It has emerged as a prime example of a multikinase network, where multiple SKs work together to detect and integrate several different signals to reach a balanced decision. The central kinase in this network, GacS, controls the phosphorylation of the RR, GacA (Fig. 1). Phosphorylated GacA activates the transcription of two non-coding RNAs, RsmY and RsmZ, and they bind and sequester the translational regulators, RsmA (Brencic *et al.*^2009^) and the more recently discovered RsmN (Morris Elizabeth *et al.*^2013^). Free RsmA and RsmN bind to certain mRNAs, promoting the degradation of transcripts involved in chronic virulence (e.g. relating to biofilm formation, T6SS and extracellular products such as pyocyanin and cyanide) while favouring those involved in acute infection (e.g. relating to T3SS and motility) (Reimmann *et al.* 1997; Parkins, Ceri and Storey 2001; Pessi *et al.*^2001^; Valverde *et al.*^2003^; Heurlier *et al.*^2004^; Burrowes *et al.*^2006^; Mulcahy *et al.* 2008; Brencic and Lory 2009; Moscoso *et al.*^2011^; Morris Elizabeth *et al.*^2013^). In short, when GacS signalling is active, GacA will be phosphorylated and this will favour the chronic mode of infection.

GacS is an unorthodox kinase (containing HisKA, HATPase, REC and Hpt domains) whose signalling activity is controlled through kinase–kinase interactions by three hybrid SKs: RetS, LadS and PA1611. RetS and LadS interact with GacS, with RetS inhibiting, and LadS activating, GacS signalling (Goodman *et al.*^2004^; Laskowski, Osborn and Kazmierczak 2004; Laskowski and Kazmierczak 2006; Ventre *et al.*^2006^). RetS downregulates GacS signalling by binding to GacS and reducing its ability to autophosphorylate (Goodman *et al.*^2009^), whereas LadS upregulates GacS signalling through a phosphorelay mechanism where phosphoryl groups are transferred from the REC domain of LadS to the Hpt domain of GacS (Chambonnier *et al.*^2016^). Unlike RetS and LadS, PA1611 does not interact with GacS; instead, PA1611 binds to RetS, which prevents it from inhibiting GacS (Kong *et al.*^2013^; Bhagirath *et al.*^2017^). The interaction of the four SKs allows for the integration of signals to modulate GacS phosphorylation levels and therefore, the output of the pathway. The signals that activate the various SKs are largely unidentified. However, GacS and RetS are controlled by molecules produced at high cell density and during the lysis of kin cells, respectively, although the identity of these molecules remains elusive (Heeb, Blumer and Haas 2002; LeRoux *et al.*^2015^). Recently, it has been shown that LadS from *P. aeruginosa* is activated by calcium ions to upregulate chronic phenotypes (Broder, Jaeger and Jenal 2016).

The importance of the GacS network has been demonstrated using infection models, with Tn-Seq studies finding that most components of the network are required in either acute and/or chronic virulence in mice (Turner *et al.*^2014^). Moreover, isolates from CF patients often have pathoadaptive mutations within GacS network components, indicating that fine-tuning the signalling of the network can facilitate long-term colonisation and bacterial survival (Cramer *et al.*^2011^; Marvig *et al.*^2015^). Interestingly, strain PA14, which was originally isolated from a burn wound, has a frameshift mutation in *ladS*. Relative to many other strains, PA14 shows enhanced acute virulence, which can, in part, be attributed to the mutation in *ladS* (Mikkelsen, McMullan and Filloux 2011). Another clinical isolate, CHA, has a deletion in *gacS* and exhibits enhanced acute virulence phenotypes (Sall *et al.*^2014^). These studies show the importance of this network in infection and how environmental pressures can reshape the virulence of *P. aeruginosa* by mutationally fine-tuning this network.

### The HptB branch of the GacS network

Two of the SKs that form part of the core of the GacS network, RetS and PA1611 (described above), also interact with HptB and together form the HptB branch of the GacS network along with two further hybrid SKs, SagS and ErcS’ (Lin *et al.*^2006^; Hsu *et al.*^2008^). HptB is a histidine phosphotransfer protein (Hpt) that serves in a phosphorelay connecting RetS, PA1611, SagS and ErcS’ with an unusual output RR, HsbR (PA3346). HsbR has an N-terminal REC domain, a protein phosphatase 2C (PP2C)-like domain and a C-terminal ser/thr kinase domain (Hsu *et al.*^2008^; Bhuwan *et al.*^2012^). When phosphorylated, HsbR acts as a phosphatase to dephosphorylate the anti-anti sigma factor, HsbA (PA3347). Dephosphorylated HsbA (red arrow, Fig. 1) then sequesters the anti-sigma factor FlgM, which is otherwise found in a complex with the sigma factor, FliA. Free FliA promotes expression of the flagellar genes and therefore both swimming and swarming motility (Bhuwan *et al.*^2012^).

When HptB is inactive (i.e. not phosphorylated or absent), the receiver domain of HsbR dephosphorylates, which causes the ser/thr kinase domain of HsbR to be more active than its phosphatase domain. Consequently, HsbR phosphorylates HsbA, preventing it from binding and sequestering FlgM. FlgM instead binds FliA and this leads to a decreased expression of the flagellar genes. Furthermore, phosphorylated HsbA (blue arrow, Fig. 1) is thought to bind to, and activate, the diguanylate cyclase HsbD, which leads to an increase in c-di-GMP and RsmY levels (Bordi *et al.*^2010^; Valentini *et al.*^2016^). How exactly HsbD modulates RsmY levels is not known, but it is known that the upregulation of *rsmY* expression in the Δ*hptB* mutant depends upon intact GacS/GacA signalling (Bordi *et al.*^2010^; Jean-Pierre, Tremblay and Deziel 2017).

### The SagS/BfiS branch of the GacS network

SagS is involved in the motile-sessile switch and resistance to antimicrobials (Petrova and Sauer 2011; Petrova *et al.*^2017^), and as well as being one of the SKs that can phosphorylate HptB (Petrova and Sauer 2011), SagS has a HptB independent signalling route. SagS regulates both RsmY and RsmZ through distinct pathways; its regulation of RsmY is HptB dependent (Bordi *et al.*^2010^; Petrova and Sauer 2011), while its regulation of RsmZ is HptB independent and involves an interaction with another SK, BfiS. BfiS is required for the transition to irreversible attachment of cells during biofilm formation. The interaction between SagS and BfiS relies upon the conserved phosphorylation sites of these SKs (Petrova and Sauer 2010, 2011). The cognate RR of BfiS, BfiR, activates expression of CafA (RNase G). CafA reduces the level of RsmZ, which is required for maturation and maintenance of biofilms (Petrova and Sauer 2010). The SagS/BfiS branch of the network, therefore, regulates the level of RsmZ post-transcriptionally, while the rest of the GacS network regulates both RsmY and RsmZ at the transcriptional level (Ventre *et al.*^2006^; Goodman *et al.*^2009^). RsmY and RsmZ levels can also be influenced by other regulators such as Anr/NarL, which downregulates both sRNAs under conditions of low oxygen, and the β-lactamase regulator, AmpR, which can upregulate RsmZ (O’Callaghan *et al.*^2011^; Balasubramanian, Kumari and Mathee 2015). It appears that levels of these sRNAs are tightly coordinated by multiple intersecting regulators to orchestrate the transition from acute to chronic virulence and the planktonic to biofilm mode of growth.

### The GacS network produces and responds to c-di-GMP

Two major ways that the GacS network is known to affect c-di-GMP levels are, first, that RsmA controls the translation of the *sadC* mRNA, which encodes the diguanylate cyclase, SadC (Moscoso *et al.*^2014^), and second, the HptB branch of the GacS network regulates the HsbD diguanylate cyclase (Valentini *et al.*^2016^). Intriguingly, in addition to controlling c-di-GMP levels, the GacS network appears to respond to c-di-GMP levels. Overexpressing diguanylate cyclases can induce the T3SS (acute) to T6SS (chronic) switch, and this is dependent upon the regulatory RNAs, RsmY and RsmZ (Moscoso *et al.*^2011^). RsmY and RsmZ levels have also been shown to be elevated in strains overexpressing diguanylate cyclases (Frangipani *et al.*^2014^). It is therefore tempting to speculate that increased c-di-GMP levels activate signalling within the GacS network to help promote biofilm formation and the chronic mode of virulence. In line with this, it has recently been shown that the PilZ domain protein, HapZ, can bind to SagS and inhibit phosphotransfer to HptB, in a c-di-GMP-dependent manner (Xu *et al.*^2016^). In addition, it is possible that c-di-GMP affects signalling elsewhere in the network in yet to be determined ways.

In summary, the GacS network is a complex multikinase network that plays a major role in deciding between acute and chronic modes of virulence, and between planktonic and biofilm modes of growth. The complexity of the network and the large number of different sensors is likely to reflect the importance of making the correct decision to the survival of the bacterium, and the need to evaluate numerous signals (e.g. Ca^2+^, kin-cell lysis, c-di-GMP plus several other as yet unidentified signals) in order to inform this decision.

## CONTROL OF CUP FIMBRIAE PRODUCTION: THE ROC NETWORK AND RCS/PVR NETWORK

Surface adhesins, known as Cup fimbriae (chaperone/usher pili), are required for the initial attachment stage of biofilm formation. *Pseudomonas aeruginosa* has three different sets of archetypal Cup fimbriae genes in its core genome (*cupA, cupB* and *cupC*). The PA14 strain has an extra set of fimbriae genes, *cupD*, within the PAPI-I pathogenicity island. The *cupB* and *cupC* genes are controlled by the Roc network, while the *cupD* genes of PA14 are regulated by the Rcs/Pvr network (Kulasekara *et al.*^2005^; Rao *et al.*^2008^; Mikkelsen *et al.*^2009^, ^2013^). In addition to regulating the CupB and CupC fimbriae, the Roc network also controls expression of the MexAB-OprM drug efflux pump (Sivaneson *et al.*^2011^).

**Figure 2.**
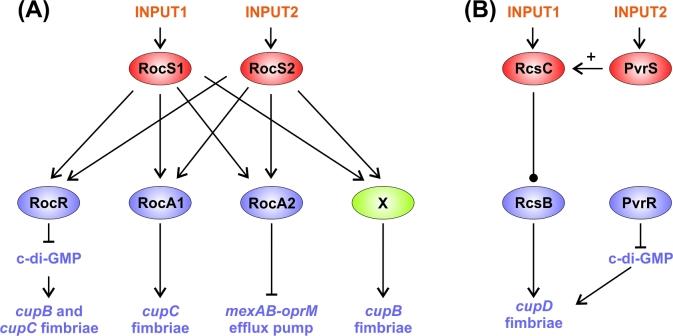
Model of the Roc network (**A**) and Rcs/Pvr network (**B**). Red ovals indicate the SKs, while the blue ovals are the RRs. The green oval is the unknown component that regulates *cupB* fimbriae. Arrows specify positive interactions and blunt-ended lines show inhibitory interactions. The bulb-ended line indicates that RcsC can have either stimulatory or inhibitory effects on RcsB depending on conditions.

Like the GacS network, the Roc network is another good example of a multikinase network, and again c-di-GMP signalling is involved, but unlike the GacS network, which is built from kinase–kinase interactions, the Roc network is instead based upon SKs sharing the same RRs (Fig. 2A). This network comprises two SKs—RocS1 and RocS2, which are both unorthodox (having HisKA, HATPase, REC and Hpt domains)—that control at least three RRs: RocA1 (helix-turn-helix DNA binding output domain), RocR (EAL, c-di-GMP degrading, phosphodiesterase output domain) and RocA2 (helix-turn-helix DNA-binding output domain). Each of the two SKs is capable of interacting with each of the RRs. The RRs target different genes: RocA1 activates expression of the CupC fimbriae, RocA2 inhibits expression of the MexAB-OprM drug efflux pump, and RocR by reducing c-di-GMP levels reduces expression of both *cupB* and *cupC* fimbriae genes. There is good reason to believe that an additional RR is involved in this network as the two SKs, RocS1 and RocS2, promote expression of CupB fimbriae genes in a manner independent of any of the three known RRs (Kulasekara *et al.*^2005^; Rao *et al.*^2008^; Sivaneson *et al.*^2011^). Although the controlling stimuli are unknown for the Roc network, the cross-regulation within this network should allow multiple inputs to be evaluated and for these signals to be integrated.

Roc network signalling promotes adhesion and therefore biofilm formation, while reducing expression of the MexAB-OprM antibiotic efflux pump. Initially, this seems counterintuitive, as biofilms are usually associated with increased antibiotic resistance. However, reduced expression of *mexAB-oprM* is seen in mature biofilms, and strains isolated from CF patients often show inactivation of this efflux pump despite having a high propensity for biofilm formation (De Kievit *et al.*^2001^; Vettoretti *et al.*^2009^). This suggests that the MexAB-OprB drug efflux pump is not involved in the antibiotic resistance of biofilms.

The *cupD* cluster, found in strain PA14, is regulated by an orthologous system to the Roc network consisting of two SKs, RcsC (unorthodox) and PvrS (hybrid), and two RRs, RcsB and PvrR (Fig. 2B). Like the Roc system, RcsB has a HTH DNA-binding domain, while PvrR has an EAL output domain. Interestingly, in this system, PvrS appears to act as a kinase, while RcsC functions primarily as a phosphatase and also acts in an intermolecular phosphorelay connecting PvrS with the output RRs. In this phosphorelay, phosphoryl groups are passed from the REC domain of the hybrid SK, PvrS, to the Hpt domain of RcsC and from there onto the REC domains of the output RRs. This kinase–kinase phosphorelay mode of interaction is reminiscent of the GacS/LadS interaction in the GacS network and is likely to represent a conserved signalling route where the Hpt domain of an unorthodox kinase is used to connect hybrid kinases (that lack Hpt domains) with their output RRs (Mikkelsen *et al.*^2009^, ^2013^; Chambonnier *et al.*^2016^).

## THE REGULATORY NETWORK CONTROLLING THE AMINOARABINOSE MODIFICATION OF LIPOPOLYSACCHARIDE

During infection, *Pseudomonas aeruginosa* needs to evade host defences such as cationic antimicrobial peptides, and to resist any antibiotic treatments that the patient may be receiving. One major way that this can be achieved is by inducing the aminoarabinose modification of the lipid A component of the lipopolysaccharide layer. This modification reduces the negative charge on the LPS, thereby limiting its electrostatic interaction with, and the subsequent uptake of, cationic antimicrobial peptides and cationic lipopeptide antibiotics (including polymyxins such as colistin, which are often used as last-resort antibiotics in CF patients). The genes required for the modification are encoded by the *arnBCADTEF* operon and it is regulated by a sensory network comprising at least five distinct TCSs each comprising a SK and a RR: PhoQP, PmrBA, ColSR, CprSR and ParSA (Macfarlane *et al.*^1999^; Macfarlane, Kwasnicka and Hancock 2000; McPhee, Lewenza and Hancock 2003; Moskowitz, Ernst and Miller 2004; Gooderham and Hancock 2009; Gooderham *et al.*^2009^; Fernández *et al.*^2010^, ^2012^; Gutu *et al.*^2013^; Lee and Ko 2014).

**Figure 3.**
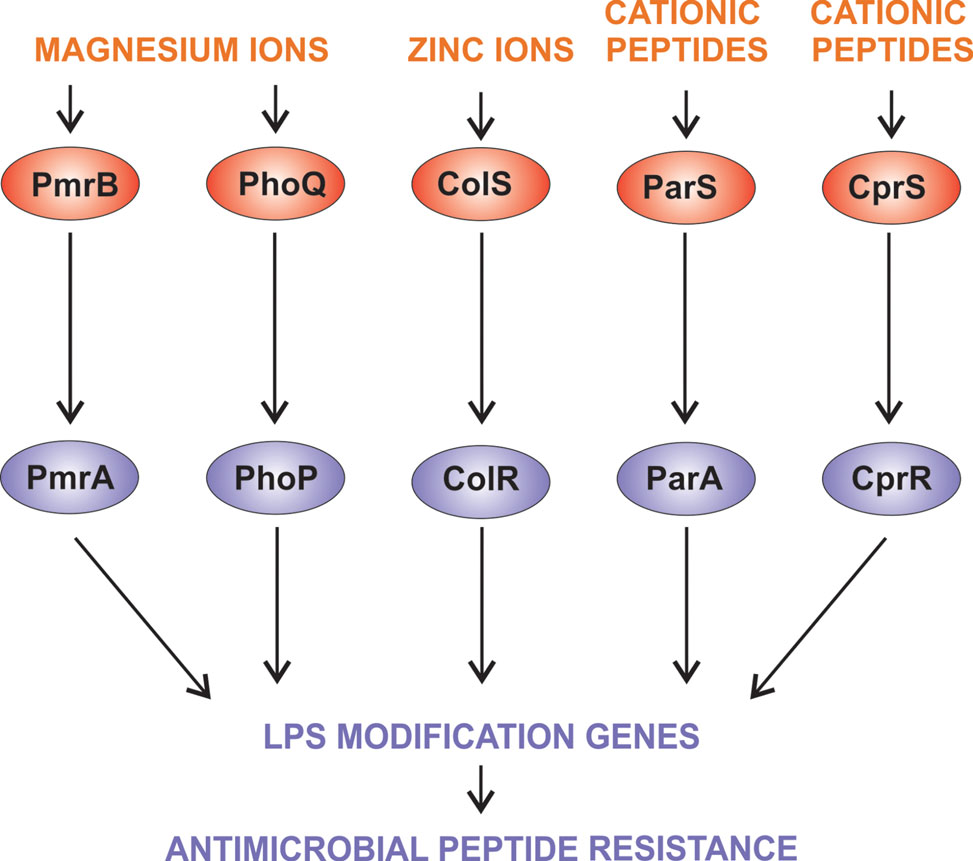
The network controlling the aminoarabinose modification of lipid A component of lipopolysaccharide. Five TCSs work together to sense magnesium ions, zinc ions and cationic antimicrobial peptides to regulate the expression of the *arnBCADTEF* operon which encodes the LPS modification enzymes. The LPS modification enhances resistance to host-derived cationic antimicrobial peptides and to polymyxin antibiotics.

Unlike the GacS and Roc networks, there is no documented linkage at the phosphosignalling level between these TCSs, instead the output RRs of the separate TCSs converge upon the aminoarabinose modification genes (Fig. 3), as a common feature of each RR's unique wider regulon. The SKs, PhoQ and PmrB, are active when the Mg^2+^ concentration is low (McPhee *et al.*^2006^), while the SKs, CprS and ParS, are activated by different cationic antimicrobial peptides (Fernández *et al.*^2010^, ^2012^; Muller, Plésiat and Jeannot 2011), and ColS is activated by Zn^2+^ (Nowicki *et al.*^2015^). Extracellular DNA is a significant component of the biofilm matrix and is often found at infection sites, and it appears to play an important physiological role in the PhoQP and PmrBA responses, as it sequesters cations and can reduce Mg^2+^ levels to the extent that PhoQ and PmrB signalling are activated, thereby promoting LPS modification and increasing resistance to host cationic peptides and polymyxins (Mulcahy, Charron-Mazenod and Lewenza 2008; Gellatly *et al.*^2012^; Lewenza 2013).

This regulatory network undergoes strong selective pressures in CF patients and adaptive mutations are frequently identified in isolates from CF patients, particularly those who have been treated with polymyxins. These mutations can be in any of the TCSs of this network although mutations affecting PhoQP and PmrBA are particularly common; generally, they lead to either increased or constitutive expression of the genes for the aminoarabinose modification, and are frequently accompanied by other mutations in non-TCS genes (such as those for LPS biogenesis and outer membrane protein assembly) that further boost resistance levels (Barrow and Kwon 2009; Fernández *et al.*^2010^; Miller *et al.*^2011^; Gellatly *et al.*^2012^; Moskowitz *et al.*^2012^; Gutu *et al.*^2013^; Jochumsen *et al.*^2016^).

## SURFACE SENSING: THE WSP CHEMOSENSORY PATHWAY

**Figure 4.**
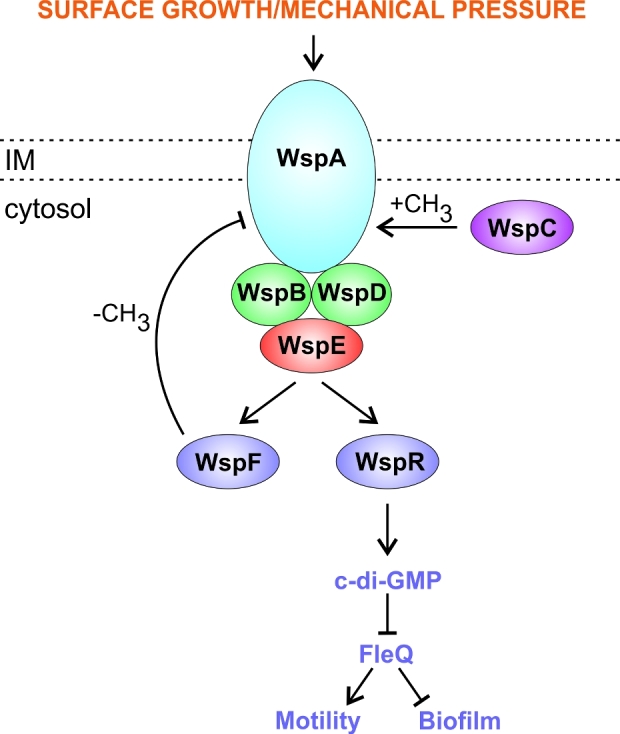
The Wsp chemosensory pathway. The proteins involved in the pathway are a methyl-accepting protein (WspA), CheW homologues (WspB and WspD), a CheA homologue (WspE), a diguanylate cyclase RR (WspR), a methylesterase RR (WspF) and a methyltransferase (WspC). Mechanical pressure associated with surface growth activates WspA, which promotes the autophosphorylation of WspE. WspE phosphorylates its two RRs, WspR and WspF. Phosphorylated WspR catalyses the synthesis of c-di-GMP (the secondary messenger output of this system). Meanwhile, phosphorylated WspF acts to reset the system by removing methyl groups from WspA, reducing its ability to activate WspE. The methylesterase activity of WspF is opposed by the constitutive methyltransferase activity of WspC.

One way that *Pseudomonas aeruginosa* responds to growth on surfaces is by activating the Wsp chemosensory system. This pathway controls the production of the secondary messenger, c-di-GMP, which promotes biofilm formation and decreases expression of the flagellar genes. Like the Chp chemosensory system (below), the Wsp chemosensory system forms a signal transduction system (Fig. 4) resembling the bacterial chemotaxis system (He and Bauer 2014). The Wsp pathway incorporates the cytoplasmic SK, WspE, which phosphorylates two RRs, the methylesterase, WspF, and the diguanylate cyclase, WspR (Bantinaki *et al.*^2007^). Surface growth is sensed by the membrane-bound WspA protein (a methyl-accepting-chemotaxis protein homologue), possibly via mechanical sensing of physical pressure resulting from surface association and cell–cell contact (O’Connor *et al.*^2012^). Contact sensing by WspA triggers autophosphorylation of WspE, which in turn phosphorylates and activates WspR and WspF. WspR-P catalyses the production of c-di-GMP through its GGDEF domain (Bantinaki *et al.*^2007^; De *et al.*^2008^, ^2009^). When WspR is dephosphorylated, it is delocalised within the cytoplasm, but when phosphorylated, it aggregates to form cytoplasmic clusters (Guvener and Harwood 2007), where its diguanylate cyclase activity is increased (Huangyutitham *et al.*^2013^). WspF-P acts to reset the system by removing methyl groups from WspA (Hickman, Tifrea and Harwood 2005; Bantinaki *et al.*^2007^). Deletion of *wspF* results in constitutive activation of WspR (WspR-P) due to overmethylation of WspA and produces a distinctive wrinkled, small colony phenotype with enhanced biofilm formation (Hickman, Tifrea and Harwood 2005).

Activation of the Wsp pathway by surface sensing triggers an increase in c-di-GMP levels (Hickman, Tifrea and Harwood 2005; O’Connor *et al.*^2012^; Ha and O’Toole 2015). The transcriptional regulator, FleQ, is the major target for the c-di-GMP produced by the Wsp pathway. FleQ promotes expression of the flagellar genes and downregulates biofilm-associated genes (e.g. *pel* encoding exopolysaccharide biosynthesis proteins). FleQ is inhibited by binding c-di-GMP, and therefore Wsp pathway activation leads to reduced expression of the flagellar genes and increased expression of biofilm-associated genes (Hickman, Tifrea and Harwood 2005; Hickman and Harwood 2008).

Consistent with its role in promoting biofilm formation, Tn-Seq data has shown that the Wsp pathway is required for chronic wound infections in mice (Turner *et al.*^2014^). Moreover, isolates from CF patients often show pathoadaptive mutations in the Wsp pathway (Marvig *et al.*^2015^); *wspF* mutations being particularly common with their distinctive phenotype of having a rugose appearance and enhanced biofilm formation (D’Argenio *et al.*^2002^; Hickman, Tifrea and Harwood 2005; Smith *et al.*^2006^; Starkey *et al.*^2009^; Sousa and Pereira 2014; Blanka *et al.*^2015^). This indicates that the Wsp pathway is under selective pressures to affect its signalling output during long-term infection, with constitutive activation being favourable for biofilm growth and chronic infection.

## SURFACE SENSING: THE CHP/FIMS/ALGR NETWORK

**Figure 5.**
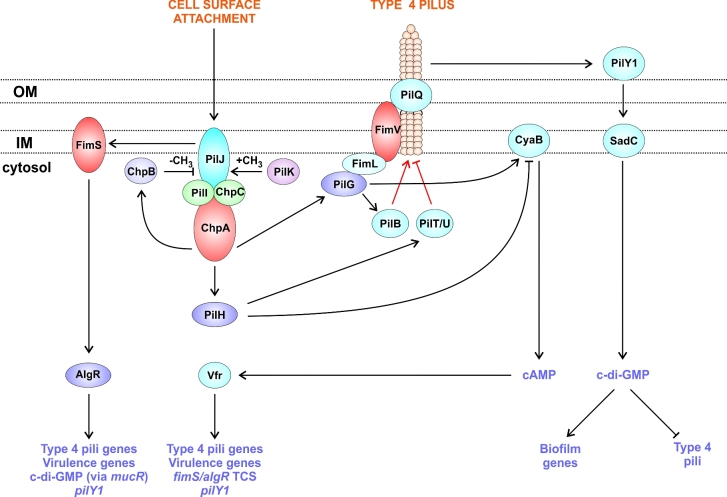
The Chp/FimS/AlgR network controls the production and operation of the type 4 pili, involved in surface attachment and twitching motility, and the expression of virulence genes. Surface contact is detected by PilJ (an MCP homologue), it activates signalling by two SKs: ChpA (a CheA homologue) and FimS. FimS phosphorylates its RR, AlgR, leading to the activation of its regulon (T4P genes, virulence genes, the diguanylate cyclase gene *mucR* and *pilY1*). ChpA phosphorylates three RRs: ChpB (a CheB homologue that mediates adaptation), PilG which activates the adenylate cyclase (CyaB) and the pilus extension ATPase (PilB), and PilH which may activate the pilus retraction ATPases (PilT/U) and inhibit adenylate cyclase (CyaB). The cAMP produced by CyaB binds to and activates the transcription factor Vfr, leading to the activation of its vast regulon, which includes T4P genes, virulence genes, the *fimS/algR* TCS and *pilY1*. After prolonged surface contact, the number of T4P increases due to AlgR and Vfr activity, which promotes the secretion of the outer-membrane surface-associated PilY1 protein. PilY1 signals to the diguanylate cyclase, SadC, which produces c-di-GMP that leads to the upregulation of biofilm genes and the downregulation of the T4P.

The Wsp pathway and the Chp/FimS/AlgR network are distinct but have many similarities; both sense surface contact, both involve a chemosensory pathway, both use secondary messenger signalling and, like many other signalling networks, both contribute to biofilm formation. In that sense they can be considered to form a super network (O’Toole and Wong 2016). The Chp/FimS/AlgR network is itself an example of a multikinase network. It regulates production of two different secondary messengers, cAMP and c-di-GMP, to control virulence and biofilm formation (Fig. 5). The production and activity of type 4 pili (T4P) are also controlled by this network and, moreover, they play a central signalling role. T4P are major surface adhesins allowing adherence and invasion of host tissues (Hahn 1997). They are located at the cell poles and undergo repeated cycles of extension, adhesion and retraction to pull the cell forward in a process called twitching motility (Skerker and Berg 2001; Mattick 2002). The extension and retraction of these pili are controlled by the Chp chemosensory pathway part of the Chp/FimS/AlgR network, which also controls levels of the secondary messenger, cyclic AMP (cAMP) (Darzins 1994; Whitchurch *et al.*^2004^; Fulcher *et al.*^2010^). cAMP regulates many other cellular processes and genes, primarily via the transcription factor Vfr (virulence factor regulator) which upregulates many virulence genes, including those involved with quorum sensing, type 2 secretion, T3SS, the FimS/AlgR TCS and the T4P themselves (Albus *et al.*^1997^; Wolfgang *et al.*^2003^; Kanack *et al.*^2006^; Bertrand, West and Engel 2010; Fulcher *et al.*^2010^).

The Chp chemosensory pathway resembles, but is distinct from, the chemotaxis pathway regulating flagellar rotation. It uses a methyl-accepting-chemotaxis-protein (MCP) homologue, PilJ, to detect surface contact and chemoattractants such as phosphatidylethanolamine (Kearns, Robinson and Shimkets 2001; Jansari *et al.*^2016^). Sensing of surface contact involves mechanosensing, where PilJ is thought to respond to tension generated within the pili, when the cell retracts pili that have adhered to surfaces (Persat *et al.*^2015^). The signal from PilJ is relayed via two adaptor proteins, PilI and ChpC, to an unorthodox SK, ChpA. ChpA is one of the most complex SKs found in any bacterial species, having nine potential phosphorylation sites; it has eight ‘Xpt’ domains, six of which are conventional Hpt domains and two that contain either serine or threonine in place of the usual phosphorylatable histidine, plus a receiver domain (ChpArec) (Whitchurch *et al.*^2004^; Leech and Mattick 2006). ChpA autophosphorylates on Hpt domains 4–6 and phosphotransfer occurs from Hpts 5 and 6 to ChpArec, but also, at a slower rate, to two standalone RRs: PilG and PilH. Reversible phosphotransfer can occur from ChpArec to Hpt 2–6; however, as yet, no phosphorylation has been observed on Hpt 1 or the remaining two ‘Xpt’ domains (Silversmith *et al.*^2016^). Hpt 2 and Hpt 3 serve as the main phosphodonors to the two output RRs, PilG and PilH (Hpt5 and 6 also contribute but at a much slower rate), that control the adenylate cyclase, CyaB (Wolfgang *et al.*^2003^; Fulcher *et al.*^2010^; Silversmith *et al.*^2016^), and the pilus extension (PilB) and retraction (PilT/U) ATPases (Bertrand, West and Engel 2010).

The RR, PilG, localises to the cell poles along with the pili forming a complex with FimL and FimV; presumably this helps to keep its local concentration high, proximal to its kinase, ChpA (Inclan *et al.*^2016^). The details of how PilG and PilH regulate adenylate cyclase and the pilus ATPases are not known, although models have been proposed based on genetic studies, where PilG stimulates pilus extension (via PilB) and CyaB activity, as the Δ*pilG* mutant has reduced piliation and reduced cAMP levels, while PilH stimulates pilus retraction (via PilT/U) and inhibits CyaB activity, as the Δ*pilF* mutant has increased piliation and increased cAMP (Bertrand, West and Engel 2010; Fulcher *et al.*^2010^). The role of PilH is controversial though, and instead it might function as a phosphate sink for PilG rather than directly regulating CyaB and PilT/U.

The Chp chemosensory pathway associates with the FimS/AlgR TCS (also known as AlgZ/R) to form the Chp/FimS/AlgR multikinase network. This network is constructed differently from the other examples of multikinase network discussed; here, the two SKs, FimS and ChpA, do not interact directly but instead they interact with a common partner, the MCP homologue, PilJ. FimS is thought to be activated by surface contact, and an attractive model would be for the surface contact sensor, PilJ, to control FimS activity via their interaction (Luo *et al.*^2015^). The FimS/AlgR TCS is best known for its role in controlling the production of the exopolysaccharide, alginate, but it is also required for twitching motility as it regulates expression of the T4P, and is involved in multiple other pathways including hydrogen cyanide and rhamnolipid production, T3SS, the Rhl quorum-sensing system and biofilm formation (Whitchurch, Alm and Mattick 1996; Okkotsu, Little and Schurr 2014).

The role of cAMP as the initial secondary messenger in the Chp/FimS/AlgR network is well known, with the Chp chemosensory system producing cAMP in response to surface contact, which activates Vfr, leading to activation of the expression of many virulence genes including the FimS/AlgR TCS. However, recently, c-di-GMP has been implicated as a delayed secondary messenger from this network (Fig. 5) i.e. following activation by surface contact, cAMP is produced initially and then several hours later c-di-GMP is produced, correlating with the onset of biofilm formation (O’Toole and Wong 2016). Two diguanylate cyclases are involved, SadC (which is also controlled by the GacS network) and MucR, with one of the targets for the c-di-GMP that they produce being the c-di-GMP binding protein, Alg44, which stimulates alginate production (Hay, Remminghorst and Rehm 2009; Schmidt *et al.*^2016^). MucR expression is stimulated by AlgR when the network senses surface contact (Kong *et al.*^2015^). Regulation of SadC is more complex; AlgR and Vfr together upregulate the *fimU*-*pilVWXXY1Y2E* operon, which is necessary for T4P biogenesis and function (Luo *et al.*^2015^). PilY1, encoded by this operon, is a cell-surface-associated protein that promotes the activity of SadC and downregulates swarming motility (Kuchma *et al.*^2010^). Crucially, PilY1 depends upon the T4P for export ensuring an ordered signalling cascade where pili are made first, before PilY1 is deployed and c-di-GMP production initiated (Luo *et al.*^2015^).

## CONCLUSIONS

TCSs play a major role in controlling *Pseudomonas aeruginosa* virulence, with over 50% of its TCSs implicated in controlling either virulence or virulence-related behaviours such as biofilm formation and antibiotic resistance (Table 1). A theme highlighted by the above examples is that during infection, *P. aeruginosa* makes extensive use of multikinase networks to detect and integrate multiple environmental signals, and to reach a balanced decision about the most appropriate response. There are a multitude of different architectures for these multikinase networks:

1. Kinase–kinase interaction. Seen in the GacS network (Fig. 1) and the Rcs/Pvr network (Fig. 2B).
2. Multiple SKs can share the same RR(s), as in the Roc network (Fig. 2A) and in the HptB branch of the GacS network (Fig. 1).
3. Connector proteins can link the SKs e.g. in the Chp/FimS/AlgR network, the surface contact sensing MCP homologue, PilJ, interacts with two SKs, ChpA and FimS (Fig. 5).
4. Regulatory convergence between TCSs, where otherwise separate TCSs control the expression of the same genes, as seen in the network controlling LPS modification (Fig. 3).
5. Transcriptional control of one TCS by another TCS e.g. in the Chp/FimS/AlgR system, the expression of the FimS/AlgR TCS is induced by Vfr, which is activated by binding cAMP that is produced by CyaB due to signalling by the ChpA SK (Fig. 5).

A further finding is that these regulatory networks undergo considerable selective pressure within hosts, particularly during chronic infection and it is common to isolate mutant strains with pathoadaptive mutations in these networks e.g. showing enhanced biofilm formation, increased antibiotic resistance or reduced motility (Marvig *et al.*^2013^, ^2015^; Jochumsen *et al.*^2016^; Winstanley, O’Brien and Brockhurst 2016). This shows that while the wild-type regulatory networks may be capable of efficiently orchestrating virulence across a broad range of conditions, there are circumstances where the networks can be genetically fine-tuned to optimise behaviour to better suit the prevailing conditions e.g. chronic infection in the CF lung, although this often comes at expense of the bacterium's ability to thrive in other conditions e.g. at causing acute infections (Smith *et al.*^2006^; Jeukens *et al.*^2014^).

Another key theme illustrated by the above examples is the interplay between multikinase networks and secondary messenger systems, with several of the networks discussed modulating levels of c-di-GMP. This provides another level of signal integration and decision making as all of the signals from several, otherwise separate, networks can feed into these secondary messengers to control common outputs important for virulence such as biofilm formation and motility.

Key priorities for the future advancement of our understanding of these multikinase networks that could facilitate the development of new ways of targeting these networks and tackling infection are as follows:

1. The ligands controlling many of the TCSs discussed above remain unknown, and although some recent progress has been made in this area (e.g. Broder, Jaeger and Jenal 2016) we urgently need systematic high-throughput methods for ligand identification.
2. Determining which kinases work together in multikinase networks is a key priority. It is likely that many of the SKs in Table 1 will feature in yet to be discovered multikinase networks. A combination of biochemical, bioinformatic and genetic methods need to be employed for systematic screening for potential interactions.
3. Revealing the complex interfaces with other regulatory mechanisms i.e. secondary messenger signalling and one-component regulators, which frequently form integral parts of multikinase networks.
4. Understanding how multikinase networks process and integrate signals to make decisions. This will require a concerted effort employing mathematical modelling alongside a detailed biochemical understanding of the regulators involved, how they respond to signal, and their interactions and expression patterns.
